# Supplementary figures and images for: Lipidomic Profiling Reveals Concerted Temporal Patterns of Functionally Related Lipids in Aedes aegypti Females Following Blood Feeding
Source: Metabolites. 2023 Mar 13;13(3):421. doi: 10.3390/metabo13030421 (PMC10051423; doi:10.3390/metabo13030421)

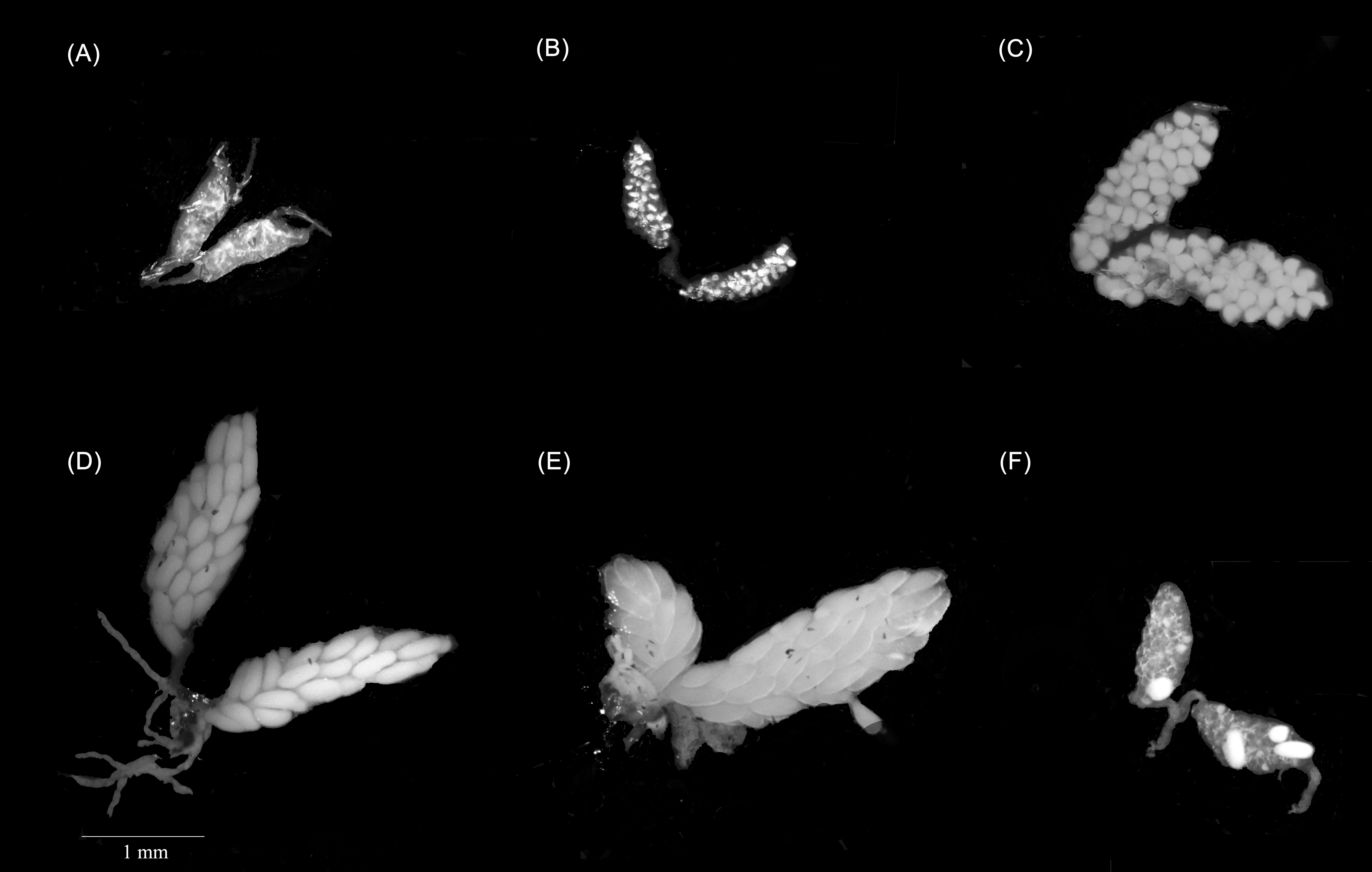

Supplement: Supplementary file 1 [file metabolites-13-00421-s001.zip › Sepplementary files/Supporting Material 1.jpg]
